# Spread of activation and deactivation in the brain: does age matter?

**DOI:** 10.3389/fnagi.2014.00288

**Published:** 2014-10-16

**Authors:** Brian A. Gordon, Chun-Yu Tse, Gabriele Gratton, Monica Fabiani

**Affiliations:** ^1^Department of Radiology, Washington University in St. LouisSt. Louis, MO, USA; ^2^Department of Psychology, Chinese University of Hong KongShatin, Hong Kong; ^3^Department of Psychology and Beckman Institute, University of IllinoisUrbana, IL, USA

**Keywords:** aging, functional magnetic resonance imaging (fMRI), task-negative network, default mode network (DMN), task-positive network, spread, activation, deactivation

## Abstract

Cross-sectional aging functional MRI results are sometimes difficult to interpret, as standard measures of activation and deactivation may confound variations in signal amplitude and spread, which however, may be differentially affected by age-related changes in various anatomical and physiological factors. To disentangle these two types of measures, here we propose a novel method to obtain independent estimates of the peak amplitude and spread of the BOLD signal in areas activated (task-positive) and deactivated (task-negative) by a Sternberg task, in 14 younger and 28 older adults. The *peak* measures indicated that, compared to younger adults, older adults had increased activation of the task-positive network, but similar levels of deactivation in the task-negative network. Measures of *signal spread* revealed that older adults had an increased spread of activation in task-positive areas, but a starkly reduced spread of deactivation in task-negative areas. These effects were consistent across regions within each network. Further, there was greater variability in the anatomical localization of peak points in older adults, leading to reduced cross-subject overlap. These results reveal factors that may confound the interpretation of studies of aging. Additionally, spread measures may be linked to local connectivity phenomena and could be particularly useful to analyze age-related deactivation patterns, complementing the results obtained with standard peak and region of interest analyses.

## INTRODUCTION

Functional MRI (fMRI) provides a powerful tool for investigating brain activity. However, inherent to many of the measures typically used for fMRI analyses, estimates of the magnitude of activation of particular voxels and of the area over which the signal spreads are conflated with one another. Changes in signal amplitude and spread may have different theoretical interpretations. While signal amplitude is supposed to reflect the degree of involvement of very precise cortical areas, signal spread may reflect the extent to which more diffuse local inhibitory or excitatory networks are involved (Tolias et al., 2005; Tehovnik et al., 2006). Importantly, separating these two properties may provide additional information to understand cognitive theories of aging. For instance, theories investigating changes in brain activity during working memory performance in aging may invoke constructs such as compensatory mechanisms that respond to increasing task difficulty (Reuter-Lorenz and Cappell, 2008; Schneider-Garces et al., 2010), which could predict a focal increase in activity, or in turn may focus on a broad loss of specialization, or dedifferentiation, of tissue (e.g., Park et al., 2004), which may lead to an increased spread of activation. Current analysis methods do not provide an accurate way to dissociate these phenomena.

Advancing age leads to cognitive decline, even in populations of healthy older adults, and it is also characterized by altered patterns of neural activity (see Kramer et al., 2006; Park and Reuter-Lorenz, 2009; Fabiani, 2012). Such changes are typically explained as up-regulation of resources, or alternatively as the reduced suppression of distracting mental processes. Importantly, these altered functional patterns greatly depend on the networks of brain areas being considered. fMRI studies indicate that during task performance, not only are some brain areas “activated” (i.e., their blood oxygen-level dependent, or BOLD, signal is higher than that observed during a baseline period), but also that others are “deactivated” (i.e., their BOLD signal is below that observed during a baseline period). For instance, attention-demanding tasks are typically associated with activation of a set of areas encompassing dorsal and lateral frontal and parietal regions forming a dorsal attentional network (DAN), with concurrent deactivation of more medial and ventral regions (the default-mode network, DMN; e.g., Shulman et al., 1997b; Mazoyer et al., 2001; Raichle et al., 2001; Raichle and Snyder, 2007). It is not clear whether aging affects focal blood flow modulations and the spread of such activations and deactivations differently. In this paper we present a novel approach whose purpose is to disambiguate peak amplitude and spread, and show how this may help understand some of the brain activation and deactivation patterns that occur with aging.

Regions within the DAN are considered to be centrally involved in controlling attention and supporting working memory and executive functions (Corbetta and Shulman, 2002). Activation of regions within the DMN has been linked to monitoring the environment (Raichle et al., 2001), stimulus-independent thoughts (Mason et al., 2007), self-referential thinking (Gusnard et al., 2001), social cognition (Harrison et al., 2008), and mental projection (Buckner and Carroll, 2007). Conversely, deactivations within the DMN during externally driven tasks suggest the suppression of these distracting mental processes, and a shift of resources to task-relevant processes (McKiernan et al., 2003, 2006; Binder et al., 2005; Sonuga-Barke and Castellanos, 2007; Castellanos et al., 2008). Failure to suppress the DMN is associated with attentional lapses (Weissman et al., 2006) and forgetting (Otten and Rugg, 2001; Daselaar et al., 2004). The DMN is strongly anti-correlated with attentional areas (Fox et al., 2005; Fransson, 2006; Toro et al., 2008) and the strength of this anti-correlation is predictive of behavioral performance on tasks requiring attentional control (Kelly et al., 2008). Thus, the literature suggests a diametric opposition and an active competition for attentional resources between these two networks (Fox et al., 2005, 2009; Fransson, 2006). For the purposes of this paper, and to avoid still-debated interpretation issues, we will label the DAN the “task-positive network,” and the DMN the “task-negative network.”

Interestingly, aging appears to impact these two networks differentially. Substantial evidence demonstrates that areas comprising the task-positive network are often up-regulated in older adults, especially during working memory and executive control studies (e.g., Jonides et al., 2000; Reuter-Lorenz et al., 2000; Cabeza et al., 2002; Grady et al., 2010; Schneider-Garces et al., 2010). In contrast, several studies find that during task performance older adults deactivate regions in the task-negative network to a lesser extent than younger adults (e.g., Lustig et al., 2003; Grady et al., 2006, 2010; Persson et al., 2007; Sambataro et al., 2010).

When examining such age-related differences, the vast majority of functional fMRI research focuses on the *amplitude* of the BOLD response. This is done using whole-brain group-level maps, or by measuring values extracted from a peak point in a region of interest (ROI). Group-level maps can be problematic as they are dependent upon spatial overlap across subjects. In cases where inter-subject topographic variability is high, different results can be obtained when examining subject-specific rather than group-level maps (see Feredoes and Postle, 2007). Further, there is an underlying assumption that the observed differences reflect variations in the magnitude of activation as a function of age rather than as a function of confounding variables that could be altering spatial properties of the BOLD signal (e.g., increased anatomical variability or smaller spread of activation).

Region of interest analyses provide the flexibility to extract signal change using a location specific to each subject or group. Still, it is not uncommon to implement a ROI peak-based approach that uses a fixed point for all subjects. A second concern relates to how the peak value is quantified. A common approach is to define ROIs using spherical kernels whose diameters can vary from quite small (3–4 mm) to quite large (8–10 mm). Such discrepancies between studies are worth further consideration, as these BOLD effects are not pure measures of amplitude. In standard ROI analyses the results are a product of both the amplitude of the signal and of how consistently that signal spreads through the volume that is being sampled. If peak amplitudes are similar, but the spread varies across two populations, drastically different results could be obtained depending on a researcher’s choice of ROI size for the measurement.

If the BOLD response around a peak is conceptualized as a Gaussian kernel, it contains two important characteristics—its height (amplitude) and its width (spread). While a great deal of the literature focuses on perceived age differences in amplitude measures, age effects on the spread of activation or deactivation have only been cursorily explored. This limited body of work usually finds that older adults have reduced spatial extents (D’Esposito et al., 1999; Buckner et al., 2000; Hesselmann et al., 2001; Huettel et al., 2001; Stebbins et al., 2002; Aizenstein et al., 2004) or that the extent varies across brain regions (Grady et al., 2010). Although intriguing, previous examinations have been limited by a combination of small sample sizes (Huettel et al., 2001; Aizenstein et al., 2004), using group rather than individual extents (Buckner et al., 2000; Cabeza et al., 2002; Mattay et al., 2002; Stebbins et al., 2002; Grady et al., 2010), approximate voxel-counting metrics (D’Esposito et al., 1999; Hesselmann et al., 2001; Huettel et al., 2001; Stebbins et al., 2002; Aizenstein et al., 2004; Sambataro et al., 2010), examining only one attentional network (all but Grady et al., 2010), or using qualitative rather than quantitative estimates of spatial extent (Cabeza et al., 2002; Grady et al., 2010).

In the papers that do take a quantitative approach, typically these “spatial extent” analyses are conducted by considering the number of voxels within a region that pass some statistical threshold-level of activation. With this approach the amplitude of the peak and its spread are confounded and no independent estimation is possible. In other words, the current literature suggests an age-related modification in the spatial extent of the BOLD signal, but such an effect has not been thoroughly explored. This is crucially important as such changes on an individual level would impact both group-level whole-brain maps, as well as the results obtained from peak ROI measures where the size of the kernel varies across studies.

Here we introduce a new quantitative approach to estimate the spatial extent, or spread, of the BOLD response (measured in mm), around peak activations and deactivations in task-positive and task-negative networks. This approach is based on estimating a parameter (*signal spread*) that reflects the rate of decay of the BOLD signal as a function of distance from its peak. As the decay is expressed *relative* to the peak, this measure does not confound signal amplitude with its spread. This spread measure could be conceptualized as reflecting how coherently local connections are engaged around peak areas. In other words, to the extent that the signal spreads further within modulated areas, it could be thought that local connections are more consistently engaged. Conversely, a reduction in spread may be associated with a loss or reduced consistency in local connections. In this way, measures of spread of the BOLD signal may provide information about the local connectivity within a particular region, separately from measures of peak amplitude, which instead are typically interpreted as estimates of the up- or down-regulation of a particular cortical region.

## MATERIALS AND METHODS

### PARTICIPANTS

The participants were 14 younger (range = 18–27; mean = 23.3; females = 6) and 28 older adults (range = 65–80; mean = 70.6; females = 12)^1^. Subjects were screened for psychological and neurological problems, medications, and vision. To participate in the experiment individuals had to be cognitively unimpaired, as indicated by score at least 51 on the modified Mini-Mental Status exam (mMMSE; Mayeux et al., 1981)^2^, and show no signs of depression on Beck’s Depression Scale (BDI; Beck et al., 1996). Participants were also administered the Vocabulary subtest of the Wechsler Adult Intelligence Scale-Revised (WAIS-R; Wechsler, 1981) and the operation word span task (O-Span, Engle et al., 1999). The university’s institutional review board approved all procedures, and participants provided written informed consent. Participants were part of a larger project and a subset of these data, involving completely independent analyses from those reported here, have been presented elsewhere (Schneider-Garces et al., 2010). Demographics are presented in **Table 1**.

**Table 1:** Demographic, behavioral, and peak variability data.

Variable	Younger adults (*N* = 14)	Older adults (*N* = 28)	*t*(40) or *F*(1,38)
Age	23.3 (2.3)	70.6 (4.3)	46.41**
Education (years)	16.0 (1.7)	16.2 (3.4)	0.40
Modified MMSE	56.7 (1.3)	55.5 (1.3)	7.84*
Vocabulary (WAIS-R)	13.0 (2.4)	13.3 (2.4)	0.15
O-Span	23.8 (9.7)	13.4 (10.1)	2.80**
Sternberg accuracy (avg)^+^	0.94 (0.05)	0.87 (0.08)	4.71*
Sternberg RT (avg)	926(176)	1068(168)	5.35*
Peak location variability (log)			
Task-positive	1.93 (0.09)	1.97 (0.02)	1.74
Task-negative	1.77 (0.08)	1.85 (0.08)	8.03*

Mean (SD); MMSE, modified mini-mental status exam; O-Span, Operation-span task. For the MMSE, Vocabulary, O-Span, and peak location variability age differences were tested using an ANCOVA in which gender and education were entered as covariates. Group significantly differ at *p < 0.05; **p < 0.005.

*^+^*F*-statistic calculated on average Fisher transformed accuracy data.*

### PROCEDURES

Subjects performed a modified version of Sternberg’s memory search task (Sternberg, 1966) with memory load varying from two to six items. Subjects saw an initial display of letters, and then had to indicate whether a subsequently presented probe was included in the array. The letters were uppercase (B, D, F, G, H, J, M, R, and T). Corresponding lower-case probes were used to avoid a direct visual match. Each letter subtended approximately 1.4° of visual angle in the diagonal. This task and design were selected because they produce robust activation of the attentional network and deactivation of the task-negative network.

The stimuli were presented across five runs. Each run consisted of five rest intervals (20 s each) and four task blocks (48 s each) alternating with each other. Within each block subjects were presented with eight trials, each beginning with the presentation of a memory set for 3 s above a fixation-cross. A 1-s maintenance interval followed where only the fixation-cross remained on screen. The probe letter was then presented for 500 ms, followed by another 1.5-s fixation-period. During this 2-s interval, subjects indicated via button press whether the probe was new or part of the preceding memory set.

Each memory set was composed of randomly chosen letters, with the constraint that no identical letters were allowed within the same set. The probe letter was present (yes response) on 50% of the trials. Load was parametrically manipulated (2, 3, 4, 5, or 6 letters) across the five runs in either ascending (2–6) or descending (6–2) order, with each run containing only one set size, yielding a total of 32 trials per load. The random assignment to an ascending or descending order was made for counterbalancing purposes and did not significantly affect either the behavioral or BOLD results.

### DATA ACQUISITION AND PREPROCESSING

Participants’ fMRI data were obtained on a Siemens Allegra 3T scanner. Data were recorded with a fast echo-planar imaging sequence with BOLD contrast (TR = 2000 ms, TE = 25 ms, flip angle = 80°, FOV = 220 mm, 64 × 64 acquisition matrix). The scans consisted of 38 slices interleaved, 3-mm-thick axial slices (3-mm in-plane resolution, 0.3-mm gap). T1-weighted anatomical scans (MPRAGE, 192 slices, 1 mm × 1 mm × 1 mm voxel size) were obtained to enable accurate anatomical coregistration.

The data were analyzed using FMRIB’s Software Library 4.1.4 (FSL; Smith, 2004; Woolrich et al., 2009). Preprocessing included motion correction, brain extraction, spatial smoothing with a Gaussian kernel of FWHM 6.0-mm, and the application of a 70-s high-pass temporal filter. Brain-extracted functional images were transformed into Montreal Neurological Institute (MNI) space through a two-stage process between the subject’s functional and T1 scans, and the subject’s T1 to the MNI template with affine transformations of 6 and 12° of freedom, respectively.

Each run was modeled as a boxcar design convolved with a gamma hemodynamic response function. Runs within a subject were combined using a fixed-effects model. Both activity positively and negatively correlated with the predicted model were considered for the current analyses. Group-level statistics were calculated using FSL with a mixed-effects design and adjusted for multiple comparisons using a cluster correction determined by *z* > 2.3 and a (corrected) cluster significance threshold of *p* = 0.05.

### ROI PEAK ANALYSES

**FIGURE 1:**
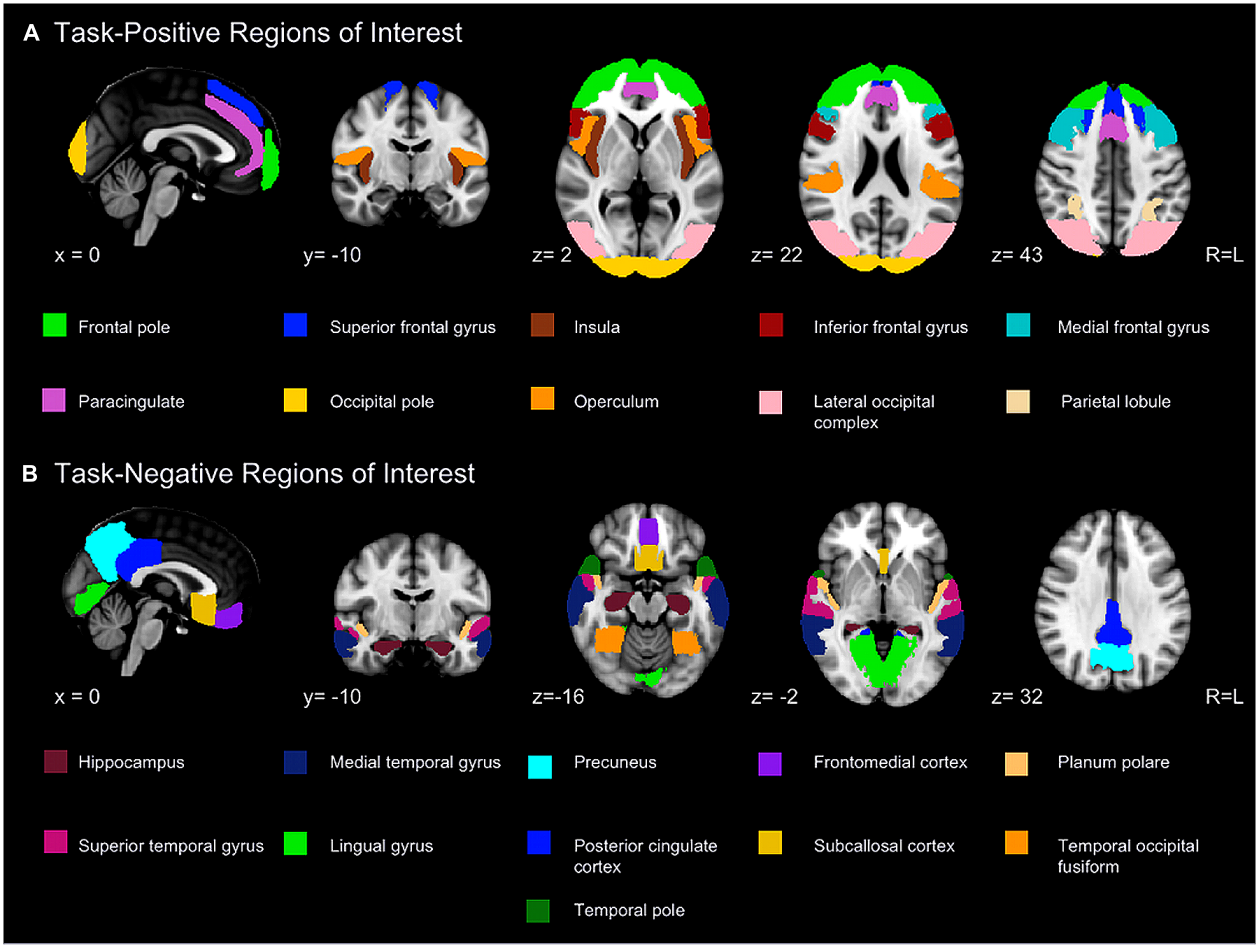
**Regions included in region of interest (ROI) analyses for the task-positive **(A)** and task-negative network (B)**.

A series of anatomical regions were selected to further examine the two networks (**Figure 1**). Peak coordinates from published studies (Shulman et al., 1997a; Raichle et al., 2001; Greicius et al., 2003; Fransson, 2005; Uddin et al., 2009) were placed into the Harvard-Oxford atlases included with FSL to select a series of ROIs. Subdivisions within a region (e.g., anterior and posterior superior temporal gyrus) were combined to yield a solitary ROI. The spatial pattern of resulting ROIs was highly congruent with visual depictions of default and attentional control networks published in the literature (Beckmann et al., 2005; Damoiseaux et al., 2006, 2008; Buckner and Carroll, 2007; Smith et al., 2009).

The ROIs were used to mask a subject’s first level analysis. In this way it was possible to extract subject- and run-specific peaks of activation or deactivation within each ROI. Allowing the peak to vary across subjects accounts for individual anatomical variability and avoids biases that might be introduced if a singular peak location was used for all subjects (Swallow et al., 2003; Devlin and Poldrack, 2007; Feredoes and Postle, 2007).

As ROIs sizes vary within the literature, a cross-section of sphere sizes was selected to determine whether different results would have been obtained with different choices of ROI sizes. Spheres with a radius of 3, 5, and 10 mm were placed around each ROI peak to assess percent signal change. Contrasts were such so that this assessed activation for the task-positive regions, and deactivation for the task-negative ones. These values were averaged across their respective networks. As the current focus is not on the load manipulation, values were also averaged across the five varying levels of difficulty. For each network and sphere size data were entered into an analysis of covariance (ANCOVA) controlling for years of education and gender.

### SPATIAL VARIABILITY

As older adults are conceptualized as being more anatomically variable due to atrophy, intra-group consistency in spatial localization is an important issue to consider (Swallow et al., 2003; Devlin and Poldrack, 2007). Within each ROI, the average Euclidian distance between each individual’s peak location in MNI space and every other subject in their group (young or old) was calculated. This captures how tightly loci of blood flow are clustered and is a measure of within group consistency in peak locations. These measures were subsequently averaged across set sizes and across all regions in a network. This resulted in every individual having one summary measure per network representing mean spatial deviation in peak locations from their respective cohort. These scores were compared between the age groups using an ANCOVA controlling for years of education and gender. Finally, a repeated-measures ANCOVA was performed examining potential network by group interactions, also controlling for education and gender.

### SPREAD OF ACTIVATION/DEACTIVATION

This measurement involved obtaining estimates of the relative amplitude of the signal at various distances from the peak point of activation or deactivation (averaged across all directions). The process of spatial smoothing acts as a spatial filter and modifies the distribution of the BOLD signal across the functional volume. As a result the BOLD data were reprocessed *without spatial smoothing*, to avoid any interaction between sphere size and smoothing kernel. Peak locations for each area and subject were then identified, and the percent signal-change values were extracted using a series of spherical ROIs with radii ranging from 3 mm (voxel size) to 10 mm, with 1-mm steps, placed around the extracted peak location. Values for the task-positive regions were obtained from the contrast positively correlated with the task, and representative of activation, while those of the task-negative regions were drawn from the negatively correlated contrast, and thus representative of deactivation. Voxels outside the brain were excluded from analysis.

Observations were then individually *normalized* for each subject, run, and ROI by dividing them by the value obtained using the 3-mm sphere. In this way subsequent measures were transformed into a proportion of the initial 3-mm sphere. This was done to account for baseline variations in the magnitude of percent signal-change data across subjects (D’Esposito et al., 2003; Ances et al., 2009). This transformation makes changes assessed with the increasing sphere sizes purely a function of the *relative* spread of the BOLD signal and therefore independent from amplitude. Values were averaged across all runs to yield one pattern per ROI per subject.

We estimated the BOLD signal changes occurring in the unique voxels added with each subsequently larger sphere (e.g., when going from 3 mm to 4 mm) using the following procedure: (a) We multiplied the volume of a sphere (e.g., 268 mm × 268 mm × 268 mm) by the normalized percent signal change value obtained with that sphere (e.g.,0.83) to compute the overall signal in each sphere adjusted for its size (e.g.,0.83^∗^268 = 222.44); (b) we subtracted the overall amount of signal change obtained for a particular sphere (e.g., 4 mm) from that obtained for the next larger sphere (e.g., 5 mm); and (c) we divided the results by the difference in volume between the two. The resulting value corresponds to the average amount of activation (or deactivation) in those *unique voxels* added when going up a step from a smaller to a larger sphere, relative to the intensity of the 3 mm sphere.

When repeated across all pairs of consecutive spheres, this analysis characterizes the decay of the BOLD response as a function of distance from the peak. This curve was then fitted using a function that assumes that signal should decay proportionally to the square of the distance from the peak (1/radius^2^) value. The slope of this relationship was considered a measure of the speed of signal “decay” around a peak point. Larger slopes correspond to a more focal pattern of activation or deactivation and faster dissipation of a signal in surrounding tissue. Conversely, smaller values as representing a slower decay or broader “spread” of the BOLD response. As the slopes were derived from “normalized” values (i.e., values relative to the peak amplitude), they should be considered as measures of spread irrespective of peak amplitude. However, we also directly examined the degree of independence of the spread and peak measures by analyzing the amount of shared variance between the two measures. Slopes were first averaged across all regions in a network and entered into an ANOVA. If these omnibus tests were significant, individual ROIs, collapsed across hemispheres, were examined.

## RESULTS

### BEHAVIORAL RESULTS

Due to a response-box malfunction, full behavioral data were unavailable for four younger adults. The mean reaction time (RT), Fisher-corrected accuracy, and Cowan’s *K* data^3^ were entered in repeated-measure ANCOVAs controlling for education and gender. The RT data indicated main effects of set size (*F*_4,136_ = 77.80, *p* < 0.001, ε = 0.7) and age group (*F*_1,34_ = 4.20, *p* < 0.05), but not a set-size by age interaction (*F*_4,136_ = 0.52, *n.s.,* ε = 0.7). Similarly, the Fisher-corrected accuracy data indicated main effects of set size (*F*_4,136_ = 8.60, *p* < 0.001, ε = 0.7) and age (*F*_1,36_ = 8.69, *p* < 0.01), but no significant interaction (*F*_4,136_ = 1.90, *p* = 0.141, ε = 0.7). Crucially, Cowan’s *K* data showed a main effect of age (*F*_1,136_ = 22.20, *p* < 0.0001), indicating that span decreased with age from 5.09 in the younger adults to 3.89 in the older adults. The average RT and accuracy data are presented in **Table 1**.

### MEAN ACTIVATION AND DEACTIVATION ANALYSES

**FIGURE 2:**
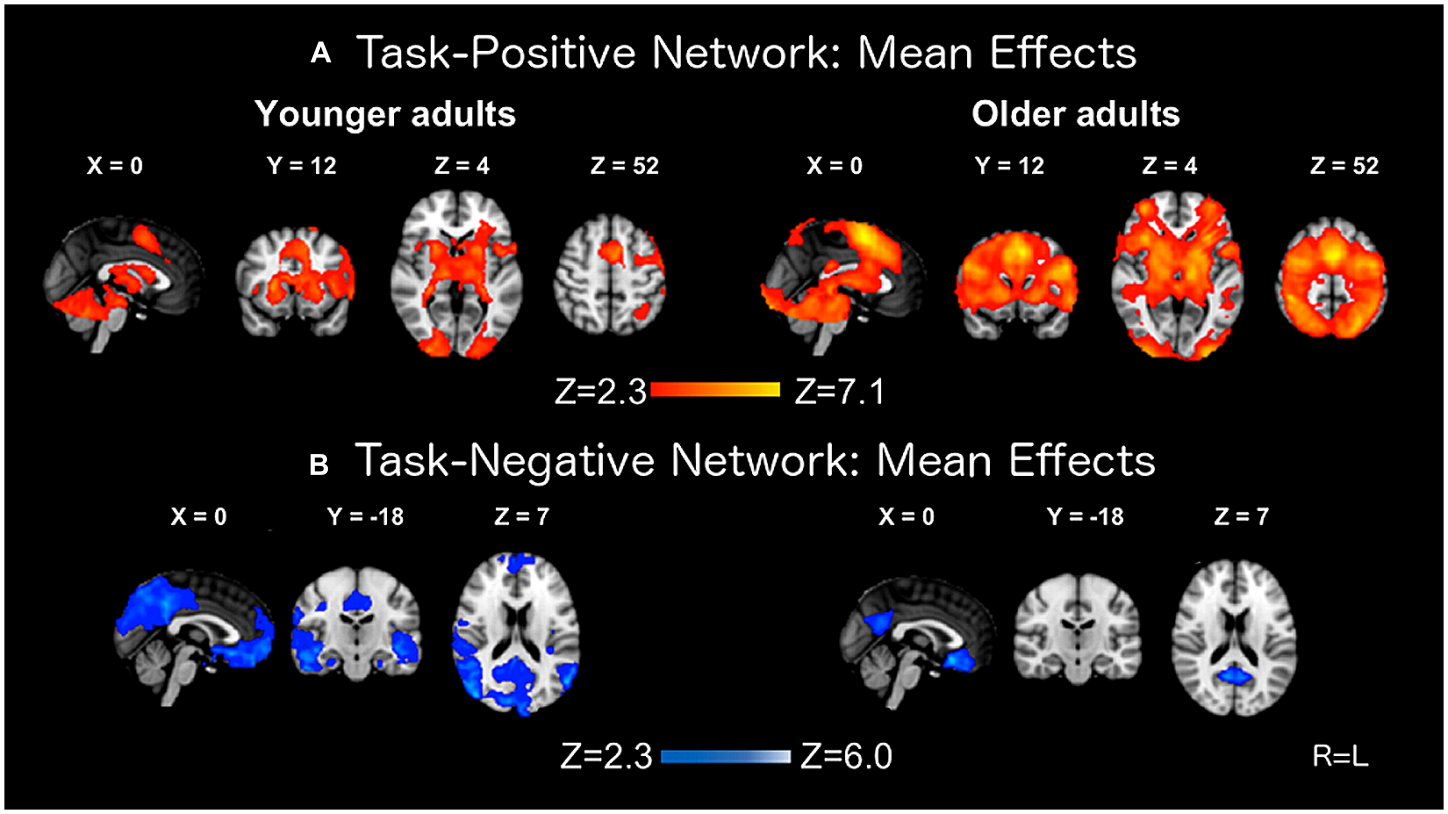
**Mean activation **(A)** and deactivation **(B)** effects on the BOLD response in younger and older adults**.

The mean contrasts for the task-positive and task-negative networks are presented in **Figure 2**. Both groups produced robust activation, with foci of recruitment in prefrontal, parietal, and occipital areas. Foci of deactivation were located in medial prefrontal cortex, precuneus, posterior cingulate cortex, medial temporal lobe, and bilateral parietal cortex. These latter brain regions are representative of areas belonging to the DMN (e.g., Raichle et al., 2001; Smith et al., 2009). Older adults showed an expanded pattern of activation, but more limited patterns of deactivation localized to medial prefrontal cortex, posterior cingulate cortex, and precuneus. These results are consistent with previous work (Lustig et al., 2003; Grady et al., 2006; Persson et al., 2007).

Whole-brain analyses were expanded by using ROI peak analyses with spheres with radii of 3, 5, and 10 mm. These values were chosen to encompass a variety of sizes that may be used in typical ROI analyses in the literature. The data were collapsed across different areas within each network, keeping task-positive and task-negative regions separate, and submitted to an ANCOVA controlling for gender and years of education. The inclusion of education did not materially alter the results of these or subsequent analyses. Education was maintained within all models to be consistent with prior work in the literature.

Grand mean values for each network and sphere size, controlling for years of education and gender, are presented in **Table 2**. Older adults had significantly greater activation of the task-positive network areas than younger adults, as measured with the 3 mm (*F*_1,38_ = 8.32, *p* < 0.01), 5 mm (*F*_1,38_ = 9.40, *p* < 0.01), and 10 mm (*F*_1,38_ = 10.30, *p* < 0.01) spheres. In contrast, older adults demonstrated significantly reduced deactivation of task-negative areas compared to younger adults only when assessed with the 10 mm sphere (*F*_1,38_ = 6.98, *p* < 0.05) but similar levels of deactivation using the 3 mm (*F*_1,38_ = 0.33, *p* = 0.57) and 5 mm (*F*_1,38_ = 0.08, *p* = 0.78) spheres. These data provide initial indication that increases in activation in the older adults are present for both the peak locations and the immediately surrounding tissue, while decreases are located only close to peak points of deactivation and rapidly weaken in surrounding tissue. This phenomenon will be examined in greater detail with the following analyses.

**Table 2:** Mean percent change values observed using 3 mm, 5 mm, and 10 mm spheres for both the task-positive and task-negative networks.

	3 mm sphere		5 mm sphere		10 mm sphere	
	Positive*	Negative	Positive**	Negative	Positive*	Negative*
Old	2.20	-1.35	1.49	-0.780	0.641	-0.14
Young	1.57	-1.25	1.02	-0.812	0.378	-0.31

Groups significantly differ at *p < 0.05 **p < 0.005.

### SPATIAL VARIABILITY

This analysis assessed the variability of the peak point across individuals and age groups. Data for this measure, controlling for years of education and gender, are presented in **Table 1**. The distribution in space of task-evoked activation peak points was not significantly different between the two age groups (*F*_1,38_ = 1.74, *p* = 0.195). Points of deactivation were significantly more variable in the older adults (*F*_1,38_ = 8.03, *p* < 0.01). The network by group interaction was not significant (*F*_1,38_ = 1.70, *p* = 0.20).

### ANALYSES OF SIGNAL SPREAD

**FIGURE 3:**
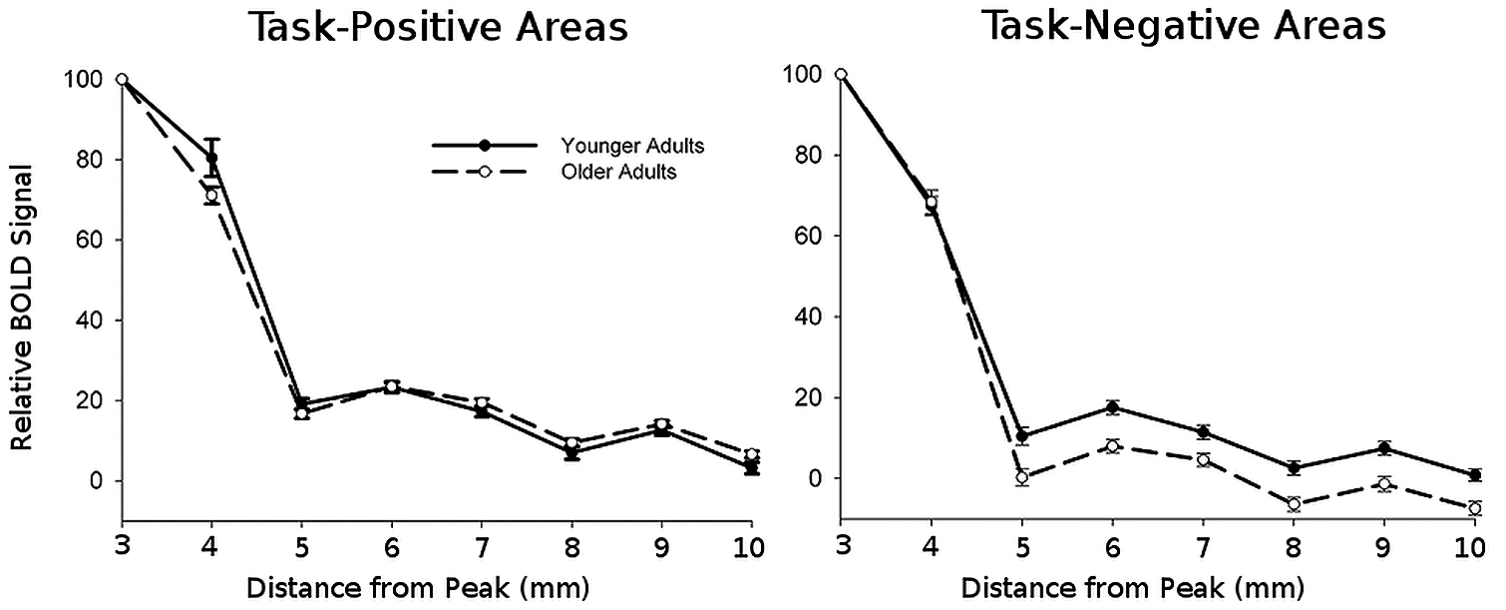
**Normalized BOLD signal amplitudes averaged across all regions within each network as a function of distance from the peak**.

To measure signal spread, we looked at the amount of task-related activation (or de-activation) present in voxels at increasing distances from peak foci. The relative signal changes for these voxels were normalized with respect to the peak value measured with a 3 mm sphere to account for variability in amplitude across subjects. Data for task-positive and task-negative networks was averaged across ROIs with a network. Group results are presented in **Figure 3**.

This figure shows that, as expected, the amplitude of the signal decays with distance. To quantify this decay, we fitted a quadratic decay function to the activation (or deactivation) values separately for each location and subject. The fits of this function with the data were typically good, with *r’*s > 0.5 in all cases^4^. The slope of this function indicates the decay/spread of the signal around the area of peak; larger values represent a more focal spread and thus a more rapid decay. All the statistical analyses were then conducted on these slope estimates. For ease of presentation, we labeled the slope of the quadratic function as “spread.”

**FIGURE 4:**
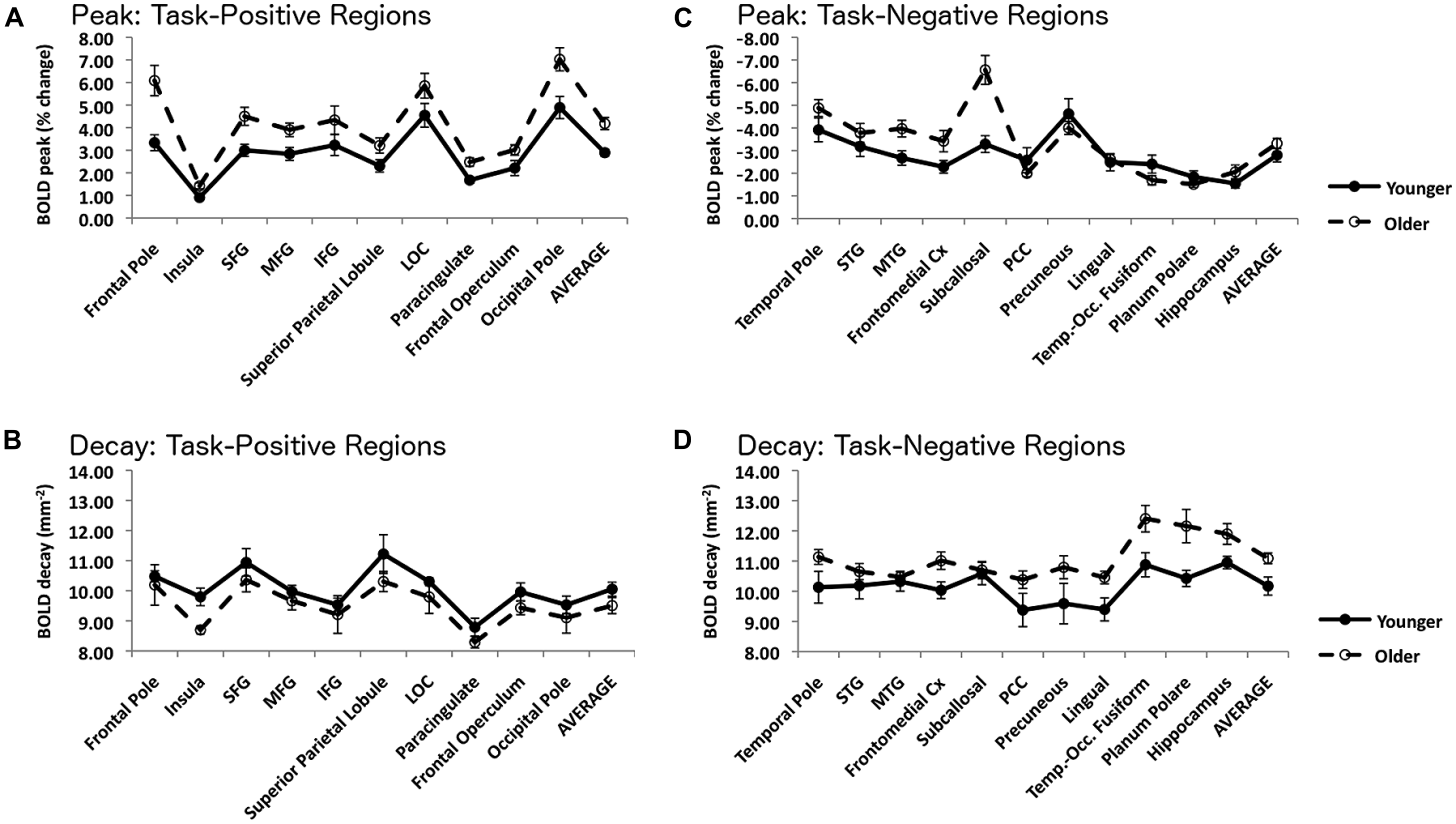
**Top left, **(A)** Amplitude of the peak BOLD signal within each ROI in the task-positive network, measured with the smaller (3 mm) kernel.** Bottom left, **(B)** Slope of the spatial decay of the BOLD signal within each ROI in the task-positive network. Top right, **(C)** Amplitude of the peak BOLD signal within each ROI in the task-negative network, measured with the smaller (3 mm) kernel. Bottom right, **(D)** Slope of the spatial decay of the BOLD signal within each ROI in the task-negative network. For all graphs error bars are based on the SE of the mean across subjects.

The decay parameters for each task-positive and task-negative area, averaged across subjects separately for younger and older adults, are presented in **Figure 4** (bottom row) and **Table 3**. The values presented are estimated grand means for task-positive and task-negative networks. The omnibus ANCOVA (controlling for education and gender) performed on data averaged across all areas and networks revealed a significant group by network interaction (*F*_1,38_ = 17.82, *p* < 0.001). Separate planned analyses for task-positive ROIs indicated a main effect of group (*F*_1,38_ = 6.99, *p* < 0.05), with the signal decaying faster in younger than older adults (left bottom graph in **Figure 4**). The opposite was true for task-negative areas (right bottom graph in **Figure 4**); the average signal decayed faster (i.e., spread less) in older than in younger adults (*F*_1,38_ = 13.82, *p* < 0.001). For both task-positive and task-negative areas, there was also a significant effect of area within a network (respectively, *F*_9,360_ = 4.00, *p* < 0.0001, and *F*_10,400_ = 4.125, *p* < 0.0001).

**Table 3:** Decay slopes for each ROI, and probability of *t*-test of the decay functions for younger and older adults.

	Decay Young	Decay Old	*p*-value
**Task positive regions**
Frontal pole	10.477	10.193	0.319
Insula	9.797	8.697	0.006
Superior frontal gyrus	10.941	10.363	0.255
Middle frontal gyrus	9.971	9.665	0.365
Inferior frontal gyrus	9.533	9.209	0.245
Superior parietal lobule	11.218	10.305	0.126
Lateral occipital complex	10.303	9.795	0.116
Paracingulate cortex	8.791	8.290	0.097
Frontal operculus	9.960	9.432	0.142
Occipital pole	9.530	9.103	0.136
**Task negative regions**
Temporal pole	10.131	11.135	0.014
Superior temporal gyrus	10.185	10.650	0.267
Middle temporal gyrus	10.321	10.473	0.626
Fronto-medial cortex	10.033	11.012	0.074
Subcallosal cortex	10.587	10.700	0.795
Posterior cingulate cortex	9.378	10.382	0.030
Precuneous	9.591	10.793	0.038
Lingual gyrus	9.398	10.456	0.005
Temporooccipital gyrus	10.875	12.403	0.023
Planum polare	10.425	12.157	0.042
Hippocampus	10.949	11.896	0.113

As the omnibus test was significant, a series of analyses examining individual ROIs within the task-positive network was performed. All regions demonstrated similar directional trends, with the younger adults having a more pronounced decay than the older adults, although this effect reached significance only in the insula (*F*_1,38_ = 9.00, *p* < 0.05). The opposite was true for task-negative areas; the average signal decayed faster (i.e., spread less) in older than in younger adults. This effect was significant in several regions, including the temporal pole (*F*_1,38_ = 6.49, *p* < 0.05, the posterior cingulate cortex (PCC; *F*_1,38_ = 5.69, *p* < 0.05), the precuneus (*F*_1,38_ = 4.29, *p* < 0.05), the lingual gyrus (*F*_1,38_ = 9.28, *p* < 0.005) and the planum polare (*F*_1,38_ = 4.40, *p* < 0.05), whereas it was marginal in fronto-medial (*F*_1,38_ = 4.02, *p* = 0.052) and temporo-occipital cortex (*F*_1,38_ = 2.92, *p* = 0.10). Thus, in general, the activation signal decayed more slowly and the deactivation signal decayed faster in older compared to younger adults.

To provide a more intuitive idea of the significance of these phenomena, we also computed the signal spread in volumetric terms. To this end, we estimated the distance at which the signal decays by 50%, and then computed the associated volume of signal spread, separately for each subject and brain region. This transformation indicates that the signal spreads to a volume that is 8.8% bigger in task-positive regions [12.45 vs. 11.44 cubic mm, t(40) = 2.667, *p* < 0.02] and 10.7% smaller in task-negative regions [9.98 vs. 11.17 cubic mm, *t*(40) = -3.48, *p* < 0.002] in the older compared to the younger adults.

#### Comparison of peak and decay/spread measures

The graphs in the top row of **Figure 4** show the peak measures obtained with the 3-mm sphere for each ROI and network. Note that this sphere size was chosen to separate the effects of peak and spread, which are confounded when using larger spheres. Note also that for the task-negative regions the most negative peak point was chosen. These graphs indicate that for the task-positive network (top left) there was a similar pattern across ROIs, with the older adults showing significantly larger peaks in the frontal pole (*F*_1,40_ = 7.78, *p* < 0.01), insula (*F*_1,40_ = 7.45, *p* < 0.01), superior frontal gyrus (*F*_1,40_ = 6.33, *p* < 0.05), middle frontal gyrus (*F*_1,40_ = 5.08, *p* < 0.05), paracingulate cortex (*F*_1,40_ = 7.89, *p* < 0.01) and occipital pole (*F*_1,40_ = 7.06, *p* < 0.05). There was also a trend in the same direction in superior parietal cortex (*F*_1,40_ = 3.10, *p* < 0.10) and frontal operculum (*F*_1,40_ = 4.07, *p* < 0.10). For the task-negative ROIs, however, the results were less consistent, with two regions showing a larger (negative) peak for older adults (subcallosal cortex: *F*_1/40_ = 12.06, *p* < 0.005; MTG: *F*_1,40_ = 5.21, *p* < 0.05), while several others showing trends in the opposite direction.

In order to test the utility of using spread measures in addition to standard measures of peak we entered peak and spread measures as simultaneous predictors in a multiple regression analysis, using age as the criterion variable. For both the task-positive and task-negative networks, the overall multiple regression results were significant [respectively, *R*(2,39) = 0.541, *p* < 0.005 for the task-positive network and *R*(2,39) = 0.512, *p* < 0.005 for the task-negative network]. For the task-positive network, the beta value was only significant for the peak measure (β = 0.431, *p* < 0.05), but not for the spread measure (β = -0.21 n.s.). For the task-negative network, the beta value was only significant for the spread measure (β = 0.452, *p* < 0.05), but not for the peak measure (β = 0.132 n.s.). This suggests that in the task-positive network the peak amplitudes are being modulated by age above and beyond changes in the spread of blood flow. Conversely in the task-negative networks there are residual age effects on the spread of deactivations after controlling for changes in amplitude.

#### Independence of peak and spread estimates

An important issue for the purposes of this paper is how independent the spread estimates are from the magnitude of the peak value. Both measures may be considered indices of the degree of cortical activation (or deactivation) during the task. It is important to know, therefore, whether they provide similar or different information. We used an intra/inter-class correlation analysis approach to assess the degree of independence of spread and peak measures. For each region we compared the average amount of variance (across subjects) that was shared between different measures. Specifically, for each region we computed four types of shared variances: (a) the average shared variance between measures of spread in one region and measures of spread in different regions of the same network (SS); (b) the average shared variance of measures of peak in one region and measures of peak in different regions of the same network (PP); (c) the average shared variance between measures of spread in one region and measures of peak in different regions of the same network (SP-all); and (d) the average shared variance between measures of spread and measures of peak taken from the same region (SP-same).

The expectation is that all correlations share the network as a common source of variance. In addition both SS and PP will have one other source of variance in common (i.e., the same type of measure), whereas SP-same will have the same region in common. SP-all correlations will have no other common sources of variance (different measures and regions) and therefore will provide an estimate of the baseline level for shared variance.

These data were submitted to a mixed-design ANOVA, with one fixed between-cases factor (network), one random factor (region, nested within network), and a four-level repeated-measure factor (correlation type). The results, averaged across task-positive, task-negative and all regions (see **Table 4**) indicated a significant effect of correlation type (*F*_3,57_ = 29.16, *p* < 0.0001). Importantly, all the intra-class correlations (i.e., PP and SS) were significant (all *F*’s_19,40_> 2.20, *p* < 0.05) even when Bonferroni-corrected. However, none of the inter-class correlations (SP, including measures of spread and peak *from the same regions*) were significant (all *F*’s_19,40_ < 1.66), with the exception of peak-spread measures for task-positive networks (*F*_19,40_ = 1.93, *p* < 0.05), which however, would not reach significance when Bonferroni-corrected. Planned comparisons showed that the same modality (PP and SS) within a network were more highly intercorrelated than measures across modalities from the same regions (SP same) (*t*_20_ = 2.39, *p* < 0.05, and *t*_20_ = 5.38, *p* < 0.001, respectively). This indicates a high degree of independence across measures. We also computed the intraclass/interclass correlations separately in younger and older adults. The results were essentially identical for the two groups. Within-measures (PP and SS) shared-variance between areas were significant (*F*_19,13_ = 4.85, *p* < 0.005 for the young group, and *F*_19,27_ = 3.44, *p* < 0.01 for the old group), whereas across-measures (SP) shared-variance between areas were not significant (*F*_19,13_ = 2.09 n.s, for the young group, and *F*_19,27_) = 0.88, n.s. for the old group). There is therefore evidence that the two measures are independent in both groups.

**Table 4:** Analysis of shared variance (intra-class correlation, *r*^2^) between spread and peak measures.

Brain networks	Spread–spread (SS)	Peak–peak (PP)	Spread–peak (SP-all)	Spread–peak (SP-same)

Shared sources of variance	Measure type network	Measure type network	Network	Region network
Task-positive	0.141	0.220	0.051	0.094
Task-negative	0.104	0.182	0.050	0.068
All regions	0.122	0.201	0.050	0.080

*p*-value vs. SP-same	0.026	0.000	0.023	

*SS = the average shared variance between measures of spread of one region and measures of spread in different regions of the same network. PP = the average shared variance between measures of peak of one region and measures of peak in different regions of the same network. SP-all = the average shared variance between measures of spread of one region and measures of peak in different regions of the same network. SP-same = the average shared variance between measures of spread and measures of peak taken from the same region.*

## DISCUSSION

The quantification of BOLD fMRI data is typically carried out on a three-dimensional volume extending over a number of voxels. As such the observed effects are not a pure measure of signal *amplitude*, but are a combination of both the peak strength of local blood flow changes as well as the *spread* of such changes throughout that volume. Anything that modulates the spread of this signal (e.g., changing the size of the smoothing kernel) can drastically impact the observed strength and localization of functional effects (see White et al., 2001; Jo et al., 2008; Mikl et al., 2008). The goal of the analyses reported in this paper was to investigate whether there were systematic age-related differences in the spreading of activation and deactivation during an attention-demanding task, and whether the spread measures provided additional information compared to the measures of peak activity.

The whole-brain analyses indicated that older adults over-activate areas positively associated with the task, while simultaneously failing to fully deactivate areas of the task-negative (DMN) network, replicating previous findings (e.g., Lustig et al., 2003; Persson et al., 2007; Park and Reuter-Lorenz, 2009). These analyses were supplemented by ROI analyses using three different sized spheres to approximate ROI analyses that are often performed in the literature. Compared to the younger adults, the older adults had greater levels of activation at all three sizes (3 mm, 5 mm, and 10 mm), but only demonstrated reduced deactivation when using the 10 mm sphere. These results are generally consistent with previous work, but also suggest that the size of the kernel used for quantification impacts the results. This is likely because, in this analysis, peak amplitude, and spread of activity are confounded.

To address this concern, we introduced a novel technique to assess the spread of activation and deactivation of the BOLD signal around its peak. This new approach shows that older adults have alterations in the *spread* of the BOLD response compared to younger adults. By measuring the *relative* (normalized) size of the BOLD response at various distances from the peak point, we could evaluate signal spread for both activation and deactivation *separately from peak amplitude*. When examining activations, the older adults had a shallower average slope of decay from the peak point. This supports previous notions that older adults possess broader (or less focused) areas of activation. Within the task-positive network this was particularly true for the insula while other regions only showed a trend for age-related differences. This suggests a systematic, but relatively subtle, increase in the spread of activations in older adults. This also suggests that the expanded activations seen in group level maps of older adults are a product of both greater peak amplitudes as well as a broader spread of such activity to surrounding tissue.

This pattern was highly significant but *inverted* for the deactivation of tissue. Older adults showed a rapid decay of deactivation with increasing distance from the peak point. This indicates that the deactivation patterns are relatively focal, and then quickly dissipate. This finding was significant across a wide range of areas including core areas of the task-negative network such as the posterior cingulate cortex, precuneus, and temporal pole. The results from this analysis indicate that older adults have a significant reduction in the spread of deactivation. They also suggest that the reduced group level deactivation maps of older adults are not due to changes in focal deactivations, but rather in how these deactivations propagate to surrounding tissue.

For voxel-wise analyses, statistics at the group level are dependent upon spatial overlap across subjects. The peak points for the older adults were significantly more spatially variable for task-negative areas. Although our analyses of the task-positive network did not reveal significant differences in spatial clustering, the numeric directions, as well as the lack of a network by group interaction, are consistent with increased spatial variability occurring throughout the brain but being more pronounced in the task-negative regions. This pattern alone would affect group-level maps, and such issues have been considered in the literature (e.g., Swallow et al., 2003; Devlin and Poldrack, 2007). This effect would compound systematic age differences in the amplitudes and spread of activations and deactivations. In areas where older adults have stronger and broader activations, such as the task-positive network, increased anatomical variability could lead to a more diffuse group-level pattern of activity. In areas where activity is narrower, such as the task-negative network, an increase in spatial variability would lead to a spatially underestimated group-level map. The type of spatial normalization could also interact with such phenomenon. By their very nature non-linear registrations warp tissue differently across the brain. Selective atrophy in aging or disease populations may exacerbate such phenomenon relative to younger adults. This could induce an artificial broadening or narrowing of spread of blood flow within a cortical region.

The general problems of spatial overlap are readily known and are a good argument in favor of ROI analyses, which provide more flexibility. Still, as seen in our typical ROI peak analyses using three different sized spheres, the choice of kernel size can interact with differences in the spread around peaks. As clearly seen in **Table 2**, the selection of a 3 or 5 mm compared to a 10 mm sphere would alter our interpretation of the data. Using the 3 or 5 mm sphere we would have concluded that the older adults had stronger activations than the younger adults in task-positive areas, while the two groups did not differ in the strength of their deactivations in task-negative areas. Using the 10 mm spheres the results would now reveal a significant group effect for both the task-positive and task-negative modulations. This does not mean that ROI analyses are inappropriate, just that interpretations must be considered in terms of both the area of tissue being modulated as well as the strength of this modulation. Such considerations are particularly important when comparing two groups that may systematically differ from each other, rather than when examining a manipulation within the same subject.

Some possible limitations to the approach proposed here should also be considered. Potential confounds when comparing younger and older adults could arise from either cortical atrophy or head motion. Atrophy would reduce the total volume that a given cortical region encompasses. Due to such shrinkage, one would expect a narrower focus where blood flow is modulated. In contrast, head motion could lead to a smearing of activity to produce a more diffuse locus of activity or deactivation. Our current data demonstrated dissociations, with older adults showing a broader extent of activity in the task-positive areas and a reduced spread in task-negative ones. As illustrated in **Figure 4**, the vast majority of regions within each network displayed consistent age-related patterns of spread despite having a range of spatial locations throughout the brain. It is highly unlikely that atrophy or head motion alone could produce such a dissociation and consistency within networks rather than manifesting as a global effect on the brain.

Another well-known concern in aging studies is the potential occurrence of age-related differences in neurovascular coupling. Neural activity is inferred from the BOLD signal based upon the relationship between neuronal firing, metabolic consumption of oxygen, and the subsequent increase in blood perfusion. The coupling between the hemodynamic response and neural activity is thought to be impaired in older age (D’Esposito et al., 1999; Buckner et al., 2000; Hesselmann et al., 2001; Huettel et al., 2001; Aizenstein et al., 2004; Fabiani et al., 2014). Most studies examine coupling in terms of activation profiles, yet the same sluggish vascular response should also impair the down-regulation of blood flow. Hence the spread of activation and deactivation should be equally (or at least similarly) impaired by a reduction in local vasculature. Therefore, the observation of a selective deficit in the deactivation spread appears inconsistent with this account.

To the extent that the patterns of activity reported here for the younger adults represent the gold standard for optimal brain function, we could speculate about possible interpretations of the alterations of signal spread in older adults. In fact, the increased spread of activation in the task-positive network in older adults is inherent to the idea of de-differentiation (Park et al., 2004) and is also consistent with notions of compensation (e.g., Cabeza et al., 2002; Persson et al., 2004; Reuter-Lorenz and Cappell, 2008; Schneider-Garces et al., 2010). It should be noted, however, that we do not mean to imply that subjects deliberately compensate for poor performance by varying the amount of spread of the activated brain areas. We are only stating that age is associated with an increase in the spread of activation in areas up-regulated during the task. The measurement approach presented in this paper may allow researchers to further explore the dissociations and overlaps existing between different models of cognitive aging.

The observation of reduced spread of deactivation in the task-negative network had not been previously characterized, and can be interpreted in several ways. Both age groups are deactivating tissue focally to the same degree, but this signal does not spread as far to neighboring tissue in the older participants. This is particularly true for core regions of the DMN such as the precuneus, posterior cingulate cortex, and fronto-medial cortex. One possible interpretation is that *local* connections are less efficient in controlling the deactivation, either mediating or possibly compounding the widely reported reduction in top-down attention control over sensory areas in older adults (e.g., Fabiani et al., 1998, 2006; Gazzaley et al., 2005, 2008).

Another interpretation is that the task-negative (DMN) network has properties that make it uniquely vulnerable to age-related declines. For example, this network has an elevated susceptibility to disrupted metabolic processes and preferentially accumulates amyloid beta (Klunk et al., 2004; Buckner et al., 2005). Reduced levels of deactivation are associated with Alzheimer’s disease (Petrella et al., 2007; Persson et al., 2008; Sperling et al., 2009) further supporting the idea that the task-negative network may be selectively linked to cognitive health. In addition, amyloid plaques in DMN regions may cause functional disruption even in older adults classified as normal (Hedden et al., 2009). Age-related structural damage in these regions may therefore be the substrate for drops in local connectivity as a function of age, which may in turn result in drops in the spread of the BOLD signal.

Although this evidence suggests that the task-negative network may be particularly sensitive to age-related decline, it should also be considered that this network is not ubiquitously less responsive in older adults (but see Grady, 2012). In a tests of emotional memory by Kensinger and Schacter (2008), older and younger adults possessed comparable levels of functional activation in regions within this network. In fact, older adults slightly over-activated these regions during encoding. Similar work using emotional stimuli has found preserved or enhanced activation in older adults (Gutchess et al., 2007). This suggests that the task-negative network is not always impaired, but rather that age differences may be more evident when it must be suppressed. It may be that older adults have difficulty inhibiting the activation of any networks, but the design of most functional studies requires a disengagement of processes that recruit the DMN during rest. Ultimately, an examination of BOLD signal spread across the task-negative network in a task that specifically activates this network, such as that reported by Grady et al. (2010), is needed. This will help determine whether the age-related effects observed in the current study are due to failing deactivation/top-down control that can affect multiple networks or if problems are specific to the task-negative network.

A third possibility is that a common mechanism may account for both the increased spread in the task-positive network and the decreased spread in the task-negative network occurring in aging. It is thought that deactivation may involve a relative inhibition or suppression of a particular cortical region. Inhibition in the cortex is carried out through GABAergic interneurons (Chagnac-Amitai and Connors, 1989) whose genetic modulators are down-regulated with age (Loerch et al., 2008; Bishop et al., 2010). Thus, a reduction in the expression of GABA receptors in the cortex may lead to a reduction of the deactivation process. The same mechanism may also account for the increased spread of the activation signal observed in older adults, as the spread of activation may be limited in younger adults by the action of inhibitory interneurons, which may be reduced in aging. This age-related change could potentially alter the balance between activation and deactivation signals in the brain, as well as the spread of these signals. Thus the same mechanism – reduction of GABAergic inhibition in the cortex – could potentially account for both reduced spread of deactivation and greater spread of activation.

Currently each proposed interpretation is plausible but speculative. It may be that no single interpretation can entirely account for these findings, but rather that a combination of multiple mechanisms drives the observed modulations in spread. For example there could be a down-regulation of GABA interneurons, but this deficit may be non-uniform across the brain. A multimodal approach combining fMRI, electrophysiological measures, and positron emission tomography tailored to the investigation of GABA receptors (e.g., Heiss and Herholz, 2006) may begin to address these questions. Improved understanding of the mechanisms that drive the changes in spread may inform studies of aging, and provide a new avenue of research to explore the brain.

In summary, many aging studies that utilize fMRI data focus on perceived differences in the amplitude of activation. Many of these analyses, particularly those drawing upon group-level maps, are actually conflating differences in amplitude with changes in the spread of blood flow. Uniquely within the field of cognitive aging, the current work independently examines both the amplitude and the spread of the BOLD response in younger and older adults. Understanding both of these properties is important when interpreting differences between these age groups. The current experiment supports previous work demonstrating over-activation and under-deactivation in older adults, while using an innovative approach to assess the spread of functional blood flow changes. This metric revealed that older adults have a broader extent of activation while simultaneously having a narrower focus of deactivation, independent of amplitude differences. These results provide a novel measure that illustrates the two-fold pattern of differences in both amplitude and spread of functional blood flow changes with increasing age.

## AUTHOR CONTRIBUTIONS

Brian A. Gordon collected the functional data, performed statistical analyses, and drafted and revised the manuscript. Chun-Yu Tse assisted in data analysis and revising the manuscript. Monica Fabiani and Gabrielle Gratton assisted in data analysis, drafting, and revising the manuscript.

## Conflict of Interest Statement

The authors declare that the research was conducted in the absence of any commercial or financial relationships that could be construed as a potential conflict of interest.

## Footnotes

1 This study was part of a multi-session project on the effects of age and fitness on neurovascular coupling. Fitness was expected to be particularly important in the older adults. For this reason, more older than younger adults were recruited. See Fabiani et al. (2014).

2 This version of the MMSE includes picture naming and forward and backward span in addition to standard questions.

3 *K* was calculated according to the method reported in Schneider-Garces et al. (2010), where these analyses are reported in a more extensive fashion.

4 We also fitted other functions – cubic, exponential — with similar, but slightly lower, fits.
